# High-Quality Genome Sequence of the Root-Knot Nematode Meloidogyne arenaria Genotype A2-O

**DOI:** 10.1128/genomeA.00519-18

**Published:** 2018-06-28

**Authors:** Kazuki Sato, Yasuhiro Kadota, Pamela Gan, Takahiro Bino, Taketo Uehara, Katsushi Yamaguchi, Yasunori Ichihashi, Noriko Maki, Hideaki Iwahori, Takamasa Suzuki, Shuji Shigenobu, Ken Shirasu

**Affiliations:** a RIKEN Center for Sustainable Resource Science, Yokohama, Kanagawa, Japan; b NIBB Core Research Facilities, National Institute for Basic Biology, Okazaki, Aichi, Japan; c Central Region Agricultural Research Center, NARO, Tsukuba, Ibaraki, Japan; d JST, PRESTO, Kawaguchi, Saitama, Japan; e Department of Agriculture, Ryukoku University, Otsu, Shiga, Japan; f Department of Biological Chemistry, College of Bioscience and Biotechnology, Chubu University, Kasugai, Aichi, Japan; g Graduate School of Science, University of Tokyo, Bunkyo, Tokyo, Japan

## Abstract

Root-knot nematodes (Meloidogyne spp.) cause serious damage to many crops globally. We report the high-quality genome sequence of Meloidogyne arenaria genotype A2-O. The genome assembly of M. arenaria A2-O is composed of 2,224 contigs with an *N*_50_ contig length of 204,551 bp and a total assembly length of 284.05 Mb.

## Abstract (precis)

Root-knot nematodes (Meloidogyne spp.) cause serious damage to many crops globally. We report the high-quality genome sequence of Meloidogyne arenaria genotype A2-O.

## GENOME ANNOUNCEMENT

Plant-parasitic nematodes are some of the most agriculturally important pests, causing estimated global losses of $80 billion per year (1). Among plant-parasitic nematodes, mitotic parthenogenetic root-knot nematodes (RKNs) (i.e., Meloidogyne incognita, Meloidogyne arenaria, and Meloidogyne javanica) are obligatory parasites which are remarkably widespread geographically (1, 2). These asexual RKNs have a broader host range and are more devastating than sexual species of RKNs (3). RKNs invade roots and induce redifferentiation of root cells to form “giant cells,” which serve as a specialized nutrient source for the parasites. The nematodes develop into adult females which lay eggs in a gelatinous matrix on the root surface.

Several genomes of agriculturally important RKN species have been sequenced using short-read sequencers (4–8). The genomes of asexual Meloidogyne species are polyploid and consist of duplicated regions with a high nucleotide divergence (∼8%) (4, 7, 8). Moreover, the genomes of asexual Meloidogyne species contain more transposable elements (TEs) than the sexual Meloidogyne hapla genome. These features might confer genomic plasticity and functional divergence between gene copies in the absence of sex and meiosis. However, these genomic features make it technically difficult to generate contiguous assemblies using short reads. In fact, the reported genome assemblies of the asexual Meloidogyne species, such as M. incognita, M. arenaria, and M. javanica, are highly fragmented compared to the M. hapla genome assembly (5, 7). To overcome this problem, we applied single-molecule real-time (SMRT) sequencing technology with the PacBio RS II platform (Pacific Biosciences, CA, USA) to sequence the genome of M. arenaria genotype A2-O, isolated in Izu Oshima Island in Japan (9). For the preparation of genomic DNA, infective second-stage juveniles of M. arenaria A2-O hatched from sterilized eggs were collected by sucrose flotation. Collected nematodes were ground down, and their genomic DNA was extracted using Genomic-tips (Qiagen, Hilden, Germany). The SMRTbell template prep kit 1.0 (Pacific Biosciences) was used to prepare 20-kb insert PacBio libraries. Then, size selection was performed with a 15-kb cutoff using Blue Pippin (Sage Science, MA, USA).

We generated an approximately 60-fold whole-genome shotgun sequence using P6-C4 chemistry on the PacBio RS II platform. A total of 754,356 reads (9.6 Gb) were assembled with Canu v1.3 (10), and the contigs were polished with Quiver (SMRT Analysis suite v2.3, Pacific Biosciences) (11). The assembled genome contains 2,224 contigs (all of the contigs are greater than 500 bp) with an *N*_50_ contig length of 204,551 bp and a total length of 284.05 Mb. The assembly was estimated to cover 94.76% of the coding space according to Core Eukaryotic Genes Mapping Approach (CEGMA) analysis (12) and is more contiguous than the previously published M. arenaria genome (7), with 14-fold fewer contigs and a 12.9-fold increased *N*_50_ contig length. This long-read-based high-quality assembly of M. arenaria should promote identification of virulence-related genes that often exist in repeat-rich or highly variable regions in the genome (13).

### Accession number(s).

The sequences obtained by this whole-genome shotgun project have been deposited in DDBJ/ENA/GenBank under the accession number QEUI00000000.
